# Breastfeeding beyond infancy

**DOI:** 10.1038/s41390-025-04235-2

**Published:** 2025-06-20

**Authors:** Neena Modi

**Affiliations:** Neonatal Medicine Imperial College London; Chelsea and Westminster NHS Foundation Trust, London, UK

## Abstract

Breast-feeding is an important issue, of relevance to individuals, societies, and national economies, hence guidance should be backed by supportive policies, a prerequisite for which is a sound evidence-base. In this commentary I discuss the findings of a paper *“*Breastfeeding beyond infancy supports adequate growth, development, and nutritional intake” by Crimmins et al. and consider the implications for policy and what next steps are warranted.

This observational study from the Arkansas Children’s Nutrition Centre in the United States addresses a very important topic, namely the evidence base for current guidelines from the American Academy of Paediatrics and the World Health Organisation, that advocate for “*continued human milk feeding, alongside the introduction of appropriate complementary foods, for twenty-four months or beyond if desired by the mother and baby*”.^1^

The authors evaluated 185 mothers and healthy, full-term children of whom 22 were exclusively formula fed (FF), 108 were “breast-fed” to 12 months of age (BF) and 55 had prolonged breast-feeding up to 24 months (PBF). Linear models were used to determine the association of feed group with anthropometrics (height, weight, head circumference), fat mass, fat free mass, and weight-for-length z-score. Maternal Body Mass Index and Intelligence Quotient (IQ), infant sex, race and age of complementary food introduction were included as covariates. The authors also assessed the association between feed group and infant development measured by the Bayley Scales of Infant Development, 3rd Edition (cognitive, language, motor, social/emotional and adaptive behaviour scales; receptive and expressive language and fine and gross motor subscales) with maternal IQ, infant sex, race and gestational age as covariates.

They found that at age 24 months, children from all groups had Bayley Scale of Infant Development scores within the normal range. There were no significant differences in weight, length, head circumference, fat mass, fat free mass, and length-for-age z-score between the groups. At 24 months, FF children had lower toddler Healthy Eating Index scores for fruit, and PBF children had higher scores for seafood compared to the other groups. There were no differences between the groups for scores for vegetables and grains. At age 24 months, PBF infants were slimmer (lower weight-for-length Z-scores), showed higher adaptive behaviour, had later complementary food introduction, and higher adherence to current national dietary guidelines. Mothers of PBF children were more likely to restrict certain foods but less likely to pressure children to eat the offered complementary foods.

The authors of this paper rightly point out that information on health outcomes in relation to prolonged breast-feeding are inconclusive. They are to be congratulated for conducting an extremely detailed study on a topic of profound global relevance and providing new information on body composition and toddler dietary intake. No detrimental effects of extended breast-feeding have been identified when accompanied by appropriate complementary food intake in this and other studies in high-income settings, and hence there appear to be no reasons to discourage a mother choosing this practice. However, as distinct from supporting maternal choice, the active promotion of extended breast feeding in global policies requires reliable evidence of a causal relationship to infant benefit. Without evidence of causality, and only on the basis of an association, policies risk targeting an intervention that will be ineffective, misleading, and a waste of resources. Thus, there is a strong association between the number of people carrying umbrellas and the likelihood of rain, but prohibiting umbrella carrying will not increase the likelihood of a sunny day. Hence it is relevant to ask if the title of the article “*Breastfeeding beyond infancy supports adequate growth, development, and nutritional intake***”** and the summary conclusion, “*extended breastfeeding beyond infancy offers continued health benefits for both mother and child and promotes sufficient growth, development and nutrition*” are justified and consider what questions remain unanswered?

The article title and summary statement imply a causal relationship between extended breast-feeding and health benefits. However, what was shown were a series of statistically significant associations, adjusted for a small set of measured confounders, and it is imprudent to conflate this unequivocally with causation. The authors rightly acknowledge the possibility that the associations may have been driven by confounders, and also, in relation to statistical significance, that “*some findings may not meet strict thresholds for multiple testing*”. Selection bias is another possibility as mothers in the PBF group had a higher IQ than those in the BP and FF groups and may have been more likely to volunteer for research study participation. Confounding by IQ would be a highly plausible explanation for the associations identified, as these mothers might be more likely to offer their toddlers seafood, more likely to engender the development of adaptive behaviours, and less likely to pressure children to eat.

A second key issue is the author’s variable use of the terms “breast-feeding” and “human milk feeding” when describing their cohort. All human milk is not the same, these terms are not interchangeable, and this lack of clarity is an important omission.^2^ Additionally, breast-feeding, defined as suckling at the breast, differs from feeding expressed breast milk by bottle. Breast-feeding, unlike feeding formula or expressed breast milk by bottle, more readily enables an infant to self-regulate intake, and the act itself represents an intimate and complex exchange between mother and infant. Mother-infant interaction plays a crucial role in infant development and as breast milk composition varies within and between feeds, self-regulation is considered an important factor in maintaining healthy growth trajectories.^3^ Pasteurised milk donated by another mother or pooled across a group of mothers, or commercial human milk products, should not be equated with milk from an infant’s own mother. Heat treatment substantially or destroys the non-nutritive factors that are considered of major importance in benefiting infant cognitive, metabolic and immune development.^4^ Nutrient, and non-nutrient content is highly variable between mothers, and influenced by genotype, diet, health, and environmental exposures. To-date, conclusive evidence of infant benefit from donor or commercial human milk products remains lacking.^5^

The importance of the topic should not be underestimated. Exclusive breast-feeding in early infancy by an infant’s own mother is recognised as a desirable goal worldwide, with benefits for both mothers and infants. Yet breast-feeding rates, even in the short-term remain stubbornly low in many countries.^6^ This is hardly surprising when globally, policies that protect women against career progression and financial penalties should she choose to breast-feed, such as universal, fully paid maternity and non-transferrable parental leave, remain insufficient.^7^ The bottom lines are that current policies are inadequate to secure exclusive breast-feeding even in the pre-weaning period, and current evidence is insufficient to formulate policies in relation to extended breast-feeding.

So, what is required to improve the evidence-base for breast-feeding duration practice and policy? The authors of the present study are correct to call for further research, but this needs to generate evidence that can justifiably impact policy development through reliable identification of causal relationships. The relevance of evidence of causality was recognised by the Nobel Committee in awarding the 2019 prize in economics to Michael Kremer, Esther Duflo and Abhijit Banerjee for driving an experimental, randomised controlled trial approach to policy development. Randomised controlled trials are considered the gold-standard approach to avoid confounding but are not ethically appropriate for many questions around breast-feeding. However, a variety of sophisticated statistical and machine learning methods to strengthen causal inferences from observational data are now available and growing rapidly, facilitated by the availability of large, routine data sources.^8^ Large data sources holding detailed, granular data, enable the application of multiple methods to address both measured (e.g., regression techniques; matching), and unmeasured, unknown (e.g., instrumental variable, difference-in-differences, and regression discontinuity analyses) confounding. Another important aspect is a priori consideration of the sample size necessary to reliably identify an effect size sufficient to influence policy. The use of routine data also facilitates population-based studies, essential to minimise selection bias. Studies conducted in multiple geographical locations improves the generalisability of findings and also can benefit from differences in confounding structures across countries. Demonstration of similar associations in locations with differing confounders increases the likelihood that a relationship is truly causal. Thus, the association between breast-feeding and child IQ holds true in both high and low-income settings but that with child obesity and blood pressure does not suggesting that the former is a causal relationship, but the latter is driven by confounders.^9^ Widespread adoption of frameworks for observational studies in which the aim is to provide evidence in support of a causal effect of an intervention would likely also be helpful in driving impact.^10^ Taken collectively, these approaches represent a practicable, achievable way forward.

The health and wellbeing of mothers and babies deserves to be more than a sound-bite. The importance of breast-feeding to population, as well as individual, health and the positive impacts on national economies, merits recognition in global policies. Questions such as whether prolonged breast-feeding affects cognitive development are vital to societies worldwide as they confront the challenges of falling birth-rates and infirm, aging populations. It is therefore to be hoped that researchers and research funders will rise to the challenge of seizing the opportunities now available, to deliver reliable answers to these fundamental breast-feeding uncertainties.
